# Supplementary material for: Modelling the health impact of food taxes and subsidies with price elasticities: The case for additional scaling of food consumption using the total food expenditure elasticity
Source: PLoS One. 2020 Mar 26;15(3):e0230506. doi: 10.1371/journal.pone.0230506 (PMC7098589; doi:10.1371/journal.pone.0230506)
Supplement: S2 Table — (DOCX) [file pone.0230506.s003.docx]

Supplementary Table 2: Standard deviations about the Marshallian cross- and own-PEs shown in Error! Reference source not found.

| Food | 1 | 2 | 3 | 4 | 5 | 6 | 7 | 8 | 9 | 10 | 11 | 12 | 13 | 14 | 15 | 16 | 17 | 18 | 19 | 20 | 21 | 22 | 23 |
| --- | --- | --- | --- | --- | --- | --- | --- | --- | --- | --- | --- | --- | --- | --- | --- | --- | --- | --- | --- | --- | --- | --- | --- |
| 1. Diet soft drinks | **0.049** | 0.053 | 0.010 | 0.014 | 0.000 | 0.001 | 0.000 | 0.000 | 0.000 | 0.000 | 0.000 | 0.000 | 0.000 | 0.000 | 0.000 | 0.000 | 0.000 | 0.000 | 0.000 | 0.000 | 0.000 | 0.000 | 0.000 |
| 1. Regular soft drinks | 0.047 | **0.051** | 0.016 | 0.021 | 0.001 | 0.001 | 0.000 | 0.000 | 0.000 | 0.000 | 0.000 | 0.000 | 0.000 | 0.000 | 0.000 | 0.001 | 0.001 | 0.000 | 0.000 | 0.001 | 0.001 | 0.001 | 0.000 |
| 1. Fruit drinks & juices | 0.006 | 0.007 | **0.045** | 0.035 | 0.001 | 0.002 | 0.000 | 0.000 | 0.000 | 0.000 | 0.000 | 0.000 | 0.000 | 0.000 | 0.000 | 0.001 | 0.001 | 0.000 | 0.001 | 0.001 | 0.001 | 0.001 | 0.000 |
| 1. Other non-alcoholic | 0.010 | 0.011 | 0.022 | **0.045** | 0.002 | 0.003 | 0.000 | 0.001 | 0.000 | 0.000 | 0.001 | 0.000 | 0.001 | 0.001 | 0.000 | 0.001 | 0.001 | 0.001 | 0.001 | 0.002 | 0.001 | 0.001 | 0.001 |
| 1. Fruit | 0.000 | 0.000 | 0.000 | 0.000 | **0.030** | 0.015 | 0.000 | 0.000 | 0.000 | 0.000 | 0.000 | 0.000 | 0.000 | 0.000 | 0.000 | 0.001 | 0.001 | 0.000 | 0.000 | 0.001 | 0.000 | 0.001 | 0.000 |
| 1. Vegetables | 0.000 | 0.000 | 0.000 | 0.000 | 0.012 | **0.039** | 0.000 | 0.001 | 0.001 | 0.000 | 0.001 | 0.000 | 0.001 | 0.001 | 0.000 | 0.001 | 0.001 | 0.001 | 0.001 | 0.001 | 0.001 | 0.002 | 0.001 |
| 1. Butter | 0.000 | 0.000 | 0.000 | 0.000 | 0.001 | 0.002 | **0.392** | 0.060 | 0.036 | 0.030 | 0.034 | 0.023 | 0.042 | 0.122 | 0.220 | 0.001 | 0.001 | 0.000 | 0.000 | 0.001 | 0.001 | 0.000 | 0.000 |
| 1. Cheese cream | 0.000 | 0.000 | 0.000 | 0.000 | 0.001 | 0.003 | 0.057 | **0.059** | 0.041 | 0.020 | 0.023 | 0.015 | 0.028 | 0.066 | 0.110 | 0.001 | 0.001 | 0.001 | 0.001 | 0.001 | 0.001 | 0.001 | 0.001 |
| 1. Ice-cream | 0.000 | 0.000 | 0.000 | 0.000 | 0.001 | 0.003 | 0.060 | 0.066 | **0.052** | 0.021 | 0.024 | 0.016 | 0.029 | 0.070 | 0.116 | 0.001 | 0.001 | 0.001 | 0.001 | 0.001 | 0.001 | 0.001 | 0.001 |
| 1. Cakes & biscuits | 0.000 | 0.000 | 0.000 | 0.000 | 0.001 | 0.002 | 0.044 | 0.030 | 0.018 | **0.043** | 0.043 | 0.036 | 0.068 | 0.049 | 0.085 | 0.001 | 0.001 | 0.001 | 0.001 | 0.001 | 0.001 | 0.001 | 0.001 |
| 1. Chocolate confectionary | 0.000 | 0.000 | 0.000 | 0.000 | 0.001 | 0.003 | 0.047 | 0.032 | 0.019 | 0.038 | **0.042** | 0.033 | 0.062 | 0.052 | 0.091 | 0.001 | 0.001 | 0.001 | 0.001 | 0.001 | 0.001 | 0.001 | 0.001 |
| 1. Pastry cook products | 0.000 | 0.000 | 0.000 | 0.000 | 0.001 | 0.002 | 0.038 | 0.026 | 0.016 | 0.045 | 0.047 | **0.044** | 0.071 | 0.042 | 0.073 | 0.001 | 0.001 | 0.000 | 0.001 | 0.001 | 0.001 | 0.001 | 0.001 |
| 1. Sauces & sugar condiments | 0.000 | 0.000 | 0.000 | 0.000 | 0.002 | 0.003 | 0.061 | 0.042 | 0.025 | 0.050 | 0.053 | 0.042 | **0.097** | 0.069 | 0.119 | 0.001 | 0.001 | 0.001 | 0.001 | 0.002 | 0.001 | 0.002 | 0.001 |
| 1. Margarine edible oil | 0.000 | 0.000 | 0.000 | 0.000 | 0.001 | 0.002 | 0.111 | 0.066 | 0.040 | 0.032 | 0.037 | 0.025 | 0.045 | **0.411** | 0.208 | 0.001 | 0.001 | 0.000 | 0.000 | 0.001 | 0.001 | 0.001 | 0.000 |
| 1. Other grocery food | 0.000 | 0.000 | 0.000 | 0.000 | 0.004 | 0.009 | 0.405 | 0.236 | 0.142 | 0.126 | 0.145 | 0.099 | 0.179 | 0.443 | **1.817** | 0.003 | 0.003 | 0.002 | 0.002 | 0.003 | 0.002 | 0.002 | 0.001 |
| 1. Fish seafood | 0.000 | 0.000 | 0.000 | 0.000 | 0.001 | 0.002 | 0.000 | 0.000 | 0.000 | 0.000 | 0.000 | 0.000 | 0.000 | 0.000 | 0.000 | **0.082** | 0.019 | 0.011 | 0.014 | 0.029 | 0.037 | 0.001 | 0.000 |
| 1. Beef lamb hogget | 0.000 | 0.000 | 0.000 | 0.000 | 0.001 | 0.002 | 0.000 | 0.001 | 0.000 | 0.000 | 0.000 | 0.000 | 0.000 | 0.000 | 0.000 | 0.019 | **0.045** | 0.031 | 0.041 | 0.019 | 0.030 | 0.001 | 0.000 |
| 1. Pork | 0.000 | 0.000 | 0.000 | 0.000 | 0.001 | 0.002 | 0.000 | 0.001 | 0.000 | 0.000 | 0.000 | 0.000 | 0.000 | 0.000 | 0.000 | 0.018 | 0.055 | **0.058** | 0.055 | 0.018 | 0.028 | 0.001 | 0.000 |
| 1. Poultry | 0.000 | 0.000 | 0.000 | 0.000 | 0.001 | 0.002 | 0.000 | 0.001 | 0.000 | 0.000 | 0.000 | 0.000 | 0.000 | 0.000 | 0.000 | 0.019 | 0.057 | 0.042 | **0.070** | 0.019 | 0.029 | 0.001 | 0.000 |
| 1. Milk yoghurt eggs | 0.000 | 0.000 | 0.000 | 0.000 | 0.001 | 0.002 | 0.000 | 0.000 | 0.000 | 0.000 | 0.000 | 0.000 | 0.000 | 0.000 | 0.000 | 0.023 | 0.015 | 0.009 | 0.011 | 0.066 | 0.028 | 0.001 | 0.000 |
| 1. Prepared processed meat | 0.000 | 0.000 | 0.000 | 0.000 | 0.001 | 0.002 | 0.000 | 0.000 | 0.000 | 0.000 | 0.000 | 0.000 | 0.000 | 0.000 | 0.000 | 0.037 | 0.029 | 0.017 | 0.021 | 0.035 | **0.181** | 0.001 | 0.000 |
| 1. Bread & breakfast cereals | 0.000 | 0.000 | 0.000 | 0.000 | 0.002 | 0.005 | 0.001 | 0.001 | 0.001 | 0.001 | 0.001 | 0.001 | 0.001 | 0.001 | 0.001 | 0.002 | 0.002 | 0.001 | 0.001 | 0.002 | 0.002 | **0.074** | 0.022 |
| 1. Pasta & other cereal | 0.000 | 0.000 | 0.000 | 0.000 | 0.002 | 0.005 | 0.001 | 0.001 | 0.001 | 0.001 | 0.001 | 0.001 | 0.001 | 0.001 | 0.001 | 0.002 | 0.002 | 0.001 | 0.001 | 0.002 | 0.002 | 0.041 | **0.067** |

Bold values on the diagonal are own-PE. The dark gray areas are the most disaggregated ‘like’ foods at which the hierarchical estimation of demand equations occurred, and the light grey areas are the next level up of food aggregation. Source: Nghiem et al (under review)[22]
