# Supplementary material for: Modelling the health impact of food taxes and subsidies with price elasticities: The case for additional scaling of food consumption using the total food expenditure elasticity
Source: PLoS One. 2020 Mar 26;15(3):e0230506. doi: 10.1371/journal.pone.0230506 (PMC7098589; doi:10.1371/journal.pone.0230506)
Supplement: S3 Table — (DOCX) [file pone.0230506.s004.docx]

Supplementary Table 3: Food group level (n=23) expenditure elasticities applied after conventional application of the PE matrix, and before the final TFEe scaling

| **Food** | **Expenditure elasticity** | **Standard deviation** |
| --- | --- | --- |
| 1. Diet soft drinks | 0.794 | 0.061 |
| 1. Regular soft drinks | 0.639 | 0.054 |
| 1. Fruit drinks & juices | 0.788 | 0.028 |
| 1. Other non-alcoholic | 0.999 | 0.040 |
| 1. Fruit | 0.816 | 0.034 |
| 1. Vegetables | 0.963 | 0.035 |
| 1. Butter | 0.282 | 0.245 |
| 1. Cheese cream | 1.585 | 0.157 |
| 1. Ice-cream | 0.628 | 0.069 |
| 1. Cakes & biscuits | 1.461 | 0.133 |
| 1. Chocolate confectionary | 1.415 | 0.124 |
| 1. Pastry cook products | 0.890 | 0.110 |
| 1. Sauces & sugar condiments | 1.718 | 0.141 |
| 1. Margarine edible oil | 0.127 | 0.157 |
| 1. Other grocery food | 1.320 | 1.089 |
| 1. Fish seafood | 0.629 | 0.062 |
| 1. Beef lamb hogget | 1.420 | 0.040 |
| 1. Pork | 1.415 | 0.054 |
| 1. Poultry | 1.165 | 0.042 |
| 1. Milk yoghurt eggs | 0.615 | 0.050 |
| 1. Prepared processed meat | 0.467 | 0.054 |
| 1. Bread & breakfast cereals | 1.009 | 0.056 |
| 1. Pasta & other cereal | 0.560 | 0.038 |
