# Supplementary material for: Modelling the health impact of food taxes and subsidies with price elasticities: The case for additional scaling of food consumption using the total food expenditure elasticity
Source: PLoS One. 2020 Mar 26;15(3):e0230506. doi: 10.1371/journal.pone.0230506 (PMC7098589; doi:10.1371/journal.pone.0230506)
Supplement: S4 Table — (DOCX) [file pone.0230506.s005.docx]

Supplementary Table 4: HALYs gained at 3% discount rate for saturated fat and sugar taxes, and fruit and vegetable subsidy, for the preferred TFEe adjustment and conventional (no TFEe adjustment) analyses

| **Change (other than baseline)** | **HALYs gained** | **95% uncertainty interval for HALYs ‡** |
| --- | --- | --- |
| ***Saturated fat tax of $2 per 100g*** |  |  |
| Naïve model – no TFEe adjustment | 904,000 |  |
| TFEe adjustment | 484,000 | (362,000 to 641,000) |
| ***Sugar tax of $0.4/100 grams per 100g*** |  |  |
| Naïve model – no TFEe adjustment | 680,000 |  |
| TFEe adjustment | 450,000 | (333,000 to 626,000) |
| ***Fruit and vegetable subsidy of 20%*** |  |  |
| Naïve model – no TFEe adjustment | -121,000 |  |
| TFEe adjustment | 252,000 | (120,000 to 356,000) |

‡ Uncertainty intervals for 2000 simulations (for TFEe adjusted results only) drawing the 2.5^th^ and 97.5^th^ percentiles.
