# Supplementary material for: Modelling the health impact of food taxes and subsidies with price elasticities: The case for additional scaling of food consumption using the total food expenditure elasticity
Source: PLoS One. 2020 Mar 26;15(3):e0230506. doi: 10.1371/journal.pone.0230506 (PMC7098589; doi:10.1371/journal.pone.0230506)
Supplement: S5 Table — (DOCX) [file pone.0230506.s006.docx]

Supplementary Table 5: Univariate sensitivity analyses about low and high (2.5th and 97.5th percentile) values of TFEe, using the “TFEe adjustment” models shown in Error! Reference source not found.

|  | **Food outputs** | | | **Health measures** | | |
| --- | --- | --- | --- | --- | --- | --- |
| **Change (other than baseline)** | **Grams of food (g.day-1)** | **Expenditure (%)** | **Energy (kJ)** | **BMI** | **HALYs gained (3% discounting)** | **HALYs gained (0% discounting)** |
| ***Saturated fat tax of $2 per 100g*** |  |  |  |  |  |  |
| Preferred model; TFE_e_ = 0.75 | -13.93 | 0.47 | -348 | -0.61 | 491,000 | 1,805,000 |
| Low TFE_e_ = 0.42 | -28.19 | 0.26 | -452 | -0.79 | 609,000 | 2,240,000 |
| High TFE_e_ = 0.96 | -4.71 | 0.61 | -280 | -0.49 | 412,000 | 1,514,000 |
| ***Sugar tax of $0.4/100 grams per 100g*** |  |  |  |  |  |  |
| Preferred model; TFE_e_ = 0.75 | -16.01 | 0.23 | -321 | -0.56 | 456,000 | 1,671,000 |
| Low TFE_e_ = 0.42 | -22.98 | 0.13 | -372 | -0.65 | 514,000 | 1,888,000 |
| High TFE_e_ = 0.96 | -11.50 | 0.29 | -288 | -0.50 | 417,000 | 1,529,000 |
| ***Fruit and vegetable subsidy of 20%*** |  |  |  |  |  |  |
| Preferred model; TFE_e_ = 0.75 | 45.33 | -0.39 | -56 | -0.10 | 258,000 | 953,000 |
| Low TFE_e_ = 0.42 | 56.34 | -0.22 | 37 | 0.07 | 133,000 | 501,000 |
| High TFE_e_ = 0.96 | 38.19 | -0.51 | -116 | -0.21 | 336,000 | 1,239,000 |
