# Supplementary material for: Modelling the health impact of food taxes and subsidies with price elasticities: The case for additional scaling of food consumption using the total food expenditure elasticity
Source: PLoS One. 2020 Mar 26;15(3):e0230506. doi: 10.1371/journal.pone.0230506 (PMC7098589; doi:10.1371/journal.pone.0230506)
Supplement: S6 Table — (DOCX) [file pone.0230506.s007.docx]

Supplementary Table 6: Univariate sensitivity analyses about low and high (2.5^th^ and 97.5^th^ percentile) values of PE disaggregation scalar

|  | **Food outputs** | | | **Health measures** | | |
| --- | --- | --- | --- | --- | --- | --- |
| **Change (other than baseline)** | **Grams of food (g.day-1)** | **Expenditure (%)** | **Energy (kJ)** | **BMI** | **QALYs gained (3% discounting)** | **QALYs gained (0% discounting)** |
| ***Saturated fat tax of $2 per 100g*** |  |  |  |  |  |  |
| Preferred model | -13.93 | 0.47 | -348 | -0.61 | 491,000 | 1,805,000 |
| 2.5th percentile | -15.31 | 0.47 | -330 | -0.58 | 469,000 | 1,724,000 |
| 97.5th percentile | -12.55 | 0.47 | -366 | -0.64 | 513,000 | 1,886,000 |
| ***Sugar tax of $0.4/100 grams per 100g*** |  |  |  |  |  |  |
| Preferred model | -16.01 | 0.23 | -321 | -0.56 | 456,000 | 1,671,000 |
| 2.5th percentile | -9.14 | 0.23 | -229 | -0.40 | 345,000 | 1,265,000 |
| 97.5th percentile | -22.90 | 0.23 | -412 | -0.72 | 562,000 | 2,065,000 |
| ***Fruit and vegetable subsidy of 20%*** |  |  |  |  |  |  |
| Preferred model | 45.33 | -0.39 | -56 | -0.10 | 258,000 | 953,000 |
| 2.5th percentile | 45.93 | -0.39 | -56 | -0.10 | 250,000 | 925,000 |
| 97.5th percentile | 44.72 | -0.39 | -56 | -0.10 | 265,000 | 981,000 |
