# Supplementary material for: Modelling the health impact of food taxes and subsidies with price elasticities: The case for additional scaling of food consumption using the total food expenditure elasticity
Source: PLoS One. 2020 Mar 26;15(3):e0230506. doi: 10.1371/journal.pone.0230506 (PMC7098589; doi:10.1371/journal.pone.0230506)
Supplement: Attachment — Submitted filename: Response to referees, Plos One 2.docx [file pone.0230506.s009.docx]

Dear Editor,

**Revisions:** **Modelling the health impact of food taxes and subsidies with price elasticities: the case for additional scaling of food consumption using the total food expenditure elasticity**

**Reviewer #1**:

The authors adopted price elasticity (PE) matrices to study food taxes. It ‘s an interesting topic and it’s useful for developing countries and areas. The authors may contribute to build the models, but the analysis in the paper is built on literatures and lack of innovation. Furthermore, the conclusions need to be more clear and concrete to show the final findings and economic meanings of the research.

***Need to improve conclusions in Abstract and Discussion***

*We have gone through the text again, looking to improve clarity. Please see multiple changes in the marked-up revision.*

**Reviewer #2**:

This paper uses previously estimated price elasticities for food products to estimate the effect on diets from a taxes on saturated fat and on sugar and a subsidy on fruits and vegetables. The paper goes on to estimate the effect of the estimated change in diets on health, in particular adjusted health years. The innovation in the paper is to adjust the price elasticities to account for the change in total expenditures on food from the change in aggregate food prices, which the authors refer to as the total food elasticity adjustment, TFEe adjustment. The authors find that the adjustment has a significant effect on the price elasticities they use to calculate changes in health.

While the author discuss the TFEe adjustment, I would have liked to have seen more detail regarding how the calculation is made. (On page 15, the authors claim that their approach is a “theoretically plausible solution.” But they don’t demonstrate that point.) If the adjustment or calculation were made using estimated share rather than price elasticities, the calculation would go something like this: Let *sit* be the share of total food expenditures *Et* in year t spent on product i. The available estimates of s assume that E is fixed. Thus is it necessary to account for the effect of price change on both s and E in order to measure the effect of the price change on total quantity q consumed of product i.

The change in q can be expressed as
Δq=s_(t+1)*E_(t+1)-s_t*E_t=Δs*E_t+s_t*ΔE+Δs*ΔE
An equivalent expression using elasticities would have been helpful.

*We agree with the referee that including the formula would be (very) helpful. Accordingly, we have added the following text to the Methods. First, an an overview of how the math is done:*

## *“Estimating post-tax or post-subsidy food quantities*

*The estimation of post-tax or post-subsidy food quantities is a two-step process. First, we propagate the food price changes through the Marshallian conditional PEs – which should result in no change in total food expenditure (due to the ‘conditional’ nature of these PEs), but is unlikely to do so for the reasons outlined above and in the Text Box. So we have to rescale all step 1 estimates to ensure this assumption is met. In the second step, we allocate the actual change in total food expenditure out across all foods using the above EEs propagated through the change in total food expenditure (given by the change in food price index and TFEe). However, this is unlikely to generate the ‘correct’ total post-tax or -subsidy food expenditure, so a further scaling step is required. THESE TWO STEPS ARE NOW OUTLINED IN DETAIL.”*

*Then we provide the formulae. (We think this is best published in the main paper, however if the Editor requires it can be moved to a ‘Methods Appendix’ and the above sentence in capitals changed to “These two steps are outlined in a Methods Appendix.”*

### *“Step 1: Application of price elasticities and scaling to ensure no change in total food expenditure*

*The before-tax or -subsidy total expenditure on food is:*

$$X^{B}=\sum_{i}^{n} q_{i}^{B}p_{i}^{B}$$

*Equation 1*

*Where* $q_{i}^{B}$*is the before tax- or -subsidy quantity and* $p_{i}^{B}$ *is the before-tax or -subsidy price per unit quantity, and i indexes foods 1, 2, 3,…,n.*

*The initial estimate of the percentage change in quantity of each food i due to price changes propagated through price elasticities is:*

$$\%\Delta q_{i, PE}=\sum_{j}^{n} \left( \%\Delta p_{j}\times\varepsilon_{ij} \right)$$

*Equation 2*

*where* $\%\Delta p_{j}$*is the percentage change in price of food j, and* $\varepsilon_{ij}$*are the food price elasticities for pairwise combinations of foods (i and j). However, it is not guaranteed that these food quantity changes when multiplied through the after-tax or -subsidy food prices will sum to the same total food expenditure before-tax or -subsidy, which is the assumption of a conditional Marshallian PE. (We need to satisfy this assumption before applying the expenditure elasticities that will ‘convert’ the calculations from conditional to unconditional.) So, we calculate the implied total food expenditure arising from Equation 2 as:*

$$X_{PE}=\sum_{i}^{n} \left( 1+\%\Delta q_{i, PE} \right) q_{i}^{B}p_{i}^{A}$$

*Equation 3*

*where* $p_{i}^{A}$ *is the after-tax or -subsidy price of each food item i. We then scale all first estimates of the percentage change in quantity to ensure no change in total food expenditure:*

$$\%\Delta q_{i, PE}^{S}=\left( \frac{X^{B}}{X_{PE}}\times\left( 1+\%\Delta q_{i, PE} \right) \right)-1$$

*Equation 4*

*And therefore:*

$$\Delta q_{i, PE}^{S}=\%\Delta q_{i, PE}^{S}\times q_{i}^{B}$$

*Equation 5*

### *Step 2: Application of expenditure elasticities and total food expenditure elasticity (TFEe) to allocate change in total food expenditure across foods*

*First, we set the change in total food expenditure as:*

$$\Delta X=\%\Delta FPI\times{TFE}_{e}\times X^{B}$$

*Equation 6*

*Where the percentage change in food price index is:*

$$\%\Delta FPI=100\%\times\left( \frac{\sum_{i}^{n} q_{i}^{B}p_{i}^{A}}{X^{B}}-1 \right)$$

*Equation 7*

*The preliminary estimate of the percentage change in food quantity due to change in total food expenditure is:*

$$\%\Delta q_{i, EE}=\%{\Delta FPI\times TFE}_{e}\times\eta_{i}$$

*Equation 8*

*where* $\eta_{i}$*the food EE of food i. (Note that Equation 8 is utilizing our assumption that total food expenditure after-tax or -subsidy is set by the* ${TFE}_{e}$ *and* $\%\Delta FPI$*.) Thus, the preliminary estimate of EE generated change in food quantity is:*

$$\Delta q_{i, EE}=\%\Delta q_{i, EE}\times(1+\%\Delta q_{i, PE}^{S})\times q_{i}^{B}$$

*The preliminary estimate of change in total food expenditure is:*

$${\Delta X}_{EE}=\sum_{i}^{n} \Delta q_{i, EE}\times p_{i}^{A}$$

*Equation 9*

*which is unlikely to equal our target change of* $\Delta X$ *(Equation 6). We therefore create a scalar:*

$$Scalar=\frac{\Delta X}{\Delta X_{EE}}$$

*Equation 10*

*and multiply all preliminary EE-generated changes in q by this scalar to generate ‘true’ after-tax or -subsidy q:*

$$q_{i}^{A}=\left( Scalar \times\Delta q_{i, EE} \right)+ \Delta q_{i, PE}^{S}+q_{i}^{B}$$

*Equation 11*

*that ensures total food expenditure post-tax or -subsidy is:*

$$X^{A}=X^{B}+\Delta X=\sum_{i}^{n} q_{i}^{A}p_{i}^{A}$$

*Equation 12”*

There is an implicit assumption that the pre-tax market price does not change in response to the taxes and subsidies. It is reasonable to assume perfect competition and constant marginal cost in food production NZ?

*The referee is correct in that we made this implicit assumption. Is it correct? We do not know, but we have no data to quantify it in either direction – hence we assume pre-market price does not change. We have added the following limitation to the Discussion:*

*“Third, we assume perfect competition in that the pre-tax market price does not vary once taxes (or subsidies) are imposed, that tax pass-through is 100%, and that the price elasticities we use (with TFEe adjustment) are reasonable proxies for long-run responses to price changes.”*

Another implicit assumption is that the estimated price elasticities apply in the long run. If I understand how the price elasticities were estimated, I believe that they reflect short run responses to price changes. But, it is expected that over time, consumers will modify their response to the initial price change. This is important since the price elasticities are used to estimate changes in diet over an individual’s lifetime .

*Correct – and see above for acknowledgement.*

If expenditures on food increase as a result of the tax/subsidy policy, might the change in nonfood spending result in health effects? For example, might there be less spending on exercise classes or health care, or less driving but more walking?

*Yes, this is possible. But it is both beyond the theoretical scope of this paper, and (likely!) well beyond the data we would have to quantify. We have not altered the manuscript.*

It would be desirable to compare the change in prices used in the research that generated the price elasticities to the implicit change in prices from the tax/subsidy policy. In particular, are the authors trying to estimate effects that are well outside the range of prices used for the price elasticity estimation?

*The experimental data we used actually mirror the modelled price changes here – we have now stated this in the paper. Thank you for pointing this oversight out. Following sentence altered:*

*“The randomized price sets used in this experiment aimed to maximize price variations on foods often suggested as targets for food subsidies or taxes (e.g. sugar tax, F&V subsidy), approximating the degree of price change we use as case studies in this paper.”*

Regarding the writing, in general, I found it difficult to understand what the authors were attempting to do and then how they did it. The paper could have been made much clearer .

*As per reviewer #1, we have gone through the text again, looking to improve clarity. Please see multiple changes in the marked-up revision.*

The paper uses estimates that were generated in other papers, for example the price elasticities. I found it difficult to follow the explanation of the price elasticity estimates. It was technical and short compared to the original papers, making it hard to obtain an intuitive understanding of the procedure. I think it might have been desirable if the discussion of how those results were derived was separated from the rest of the discussion.

*As above, we have tried to improve clarity of this paper. The main contribution of this paper is the disaggregation of food groups and the TFEe – steps beyond the ‘starting PE matrix’ itself. We have tried to make this clearer as well (see marked up Manuscript).*

There are many typos and places where the writing could be made clearer and the presentation improved. Here are some examples, but this is not a comprehensive list:

*Thank you for pointing these out – specific comments below.*

P3. HALY is defined in the abstract. It should be defined the first time it is used in the body of the paper .

*Done.*

P6, 1st paragraph. The explanation of price elasticity is very basic and could be dropped or relegated to a footnote. On the other hand the explanation for the need to scale the estimated elasticities is not well articulated .

*We have written this paper for all of economists, epidemiologist and people interested in public health nutrition. This reviewer – we suspect – is an economist. We have left the basic explanation in for those other readers.*

*The need to scale is made in the text box – we have tried to make this clearer in the revisions to aid clarity.*

P6, 2nd paragraph. The following is unclear, “…on both price and demand or purchasing with sufficient variation in price…” as is “…starting proportionate consumption…”

*Thanks. Changed to:*

*“Econometric analyses to generate PEs are demanding, requiring simultaneous data on both price and demand and (ideally) substantial variation in price (e.g. by time or by region).”*

*“But there are inevitably variations between settings in food purchasing patterns, prices and demand relationships.”*

P6, 2nd paragraph. The sentence beginning with “Indeed, the most common…” could be improved by more clearly noting that it is in reference to studies that focus just on food consumption. I first thought it meant general studies of price elasticities that assume no change in food expenditures, which didn’t make any sense .

*Thanks. Changed to:*

*“Indeed, the most common food price elasticity matrix is a ‘conditional’ one meaning that it is generated under the assumption that there is no change in total food expenditure for food price changes;… ”*

P6, 3rd paragraph. Consider the sentence, “Is applying PEs derived from one setting into another acceptable?” I found this misleading. At first I thought the task was that the paper was taking price elasticity estimated from one data source, and using them to estimate changes using a different data set. But that is not exactly what is being done .

*Thanks. We have added this sentence before the sentence beginning “Is applying….”:*

*“For example, our intended modelling will be using National Nutrition Survey (NNS) data from 2009 [21] (whilst older, it is nationally representative and has data by ethnicity and for a wide range of food groups), where the price elasticities were generated with experimental data (conducted in 2016, using foods with overlap but not exact concordance with NNS food groups)[22, 23] using Bayesian priors from price elasticities calculated using Household Economic Survey data (2007/08 and 2009/10) and Food Price Index data (2007 and 2010).[5]”*

The paper does not apply them in another “setting”. I struggled with trying to understand what the authors were doing, and found the writing confusing rather than enlightening .

*Please see above.*

P7-8. Given that the objective is to estimate the effect on consumption of food items from a price change, the suggestion that one would do assuming that energy intake or grams of food purchased is constant, makes no intuitive sense. I don’t see the point of discussing those two options .

*Whilst we have sympathy with the referee’s view (and agree!), these approaches have been used. So we would prefer to leave the text in – unless the Editor requests we delete it.*

P10, 2nd line. “more important” is not what you mean, but rather “larger” .

*Thank you – corrected.*

P13, Results. Information on the consumption of saturated fats and sugar would have been helpful in understanding how the tax results in a price increase of 3.91% for all food .

*Table 1 includes average under BAU intake of saturated fat, fruit and vegetables – but not sugar which has now been added.*

P22, Text Box. The text says a 20% subsidy, while the spreadsheets refer to a 10% subsidy .

*Thank you! We have changed 10% to 20% in the table legend descriptors for the Text Box.*

Sincerely, Tony Blakely (on behalf of authors)
